# Hearing Preservation in a Vestibular Schwannoma Patient via a Retrosigmoid Approach

**DOI:** 10.7759/cureus.18403

**Published:** 2021-09-30

**Authors:** Dillon Dejam, Kevin Ding, Courtney Duong, Vera Ong, Isaac Yang

**Affiliations:** 1 Neurosurgery, David Geffen School of Medicine, University of California, Los Angeles, Los Angeles, USA; 2 Neurological Surgery, Ronald Reagan University of California, Los Angeles, Medical Center, Los Angeles, USA

**Keywords:** hearing preservation, middle cranial fossa, retrosigmoid, vestibular schwannoma, neurosurgery

## Abstract

Vestibular schwannoma(s) (VS) are benign tumors of the cerebellopontine angle comprising the Schwann cells that line the vestibular branch of cranial nerve VIII. Treatment goals focus on the preservation and improvement of facial nerve and hearing function as well as tumor control. The retrosigmoid (RS) approach is associated with lower hearing preservation rates compared to the middle cranial fossa (MCF) approach. A 60-year-old male was diagnosed with right-sided cystic VS and subsequently underwent surgical resection via a RS approach. Although his preoperative hearing function was quite low, with a right-sided speech reception threshold of 35 dB and a right-sided word recognition score of 48%, he experienced a drastic improvement in his hearing postoperatively with stable residual tumor. Although the RS approach for VS resection is not considered to be as effective at preserving hearing function compared to the MCF approach, we present a case where it resulted in significantly improved hearing function. Additionally, in cases where preoperative hearing function is severely diminished, hearing preservation is not typically an outcome that is considered. However, this case suggests that improvement of hearing in these patients may be attainable, particularly with the RS approach.

## Introduction

Vestibular schwannoma(s) (VS) account for roughly 6%-8% of intracranial tumors and are the most common tumors of the cerebellopontine angle (CPA) [1,2]. Often recognized as slow-growing and benign lesions, they stem from an overproduction of the Schwann cells that line cranial nerve (CN) VIII [3]. VS are known to impede auditory, vestibular, and facial nerve function, and priorities for treatment focus on tumor control as well as preservation and restoration of CN function [4]. Patients often undergo conservative management if presenting with minimal symptoms, but when more significant symptoms are present, more thorough treatment modalities may be preferred [5].

Surgical strategies include the translabyrinthine (TL), middle cranial fossa (MCF), and retrosigmoid (RS) approaches. Although advantages of the TL approach include full exposure of the internal auditory canal (IAC), exposure of CN VII from the stylomastoid foramen to the brainstem, and direct access to the CPA, the major disadvantage of this approach is that hearing is sacrificed [6]. However, the MCF and RS approaches do not entirely abolish hearing function. Advantages of the RS approach include the ease of facial nerve identification and ability to resect tumors of all sizes, whereas the MCF approach’s main advantage is that it has better rates of hearing preservation when compared to the RS approach [7,8].

While the MCF approach is thought to produce the best rates of hearing preservation, we report the case of a patient whose hearing function greatly improved after undergoing the RS approach. The patient consented to the possibility of his data being used for research. All protected health information was secured according to the Health Insurance Portability and Accountability Act, and this study was approved by our Institutional Review Board.

## Case presentation

History, physical examination and course of treatment

A 60-year-old male with no pertinent past medical history was initially seen by his physician for a right ear infection and received audiometry testing. The results of the audiometry exam revealed significant hearing asymmetry, and subsequent MRI revealed a right VS.

After further evaluation with a neurosurgeon, the patient agreed to follow a treatment plan involving a right RS craniotomy for the resection of his right VS. A complex stereotactic extended RS craniotomy for a transcondylar posterior fossa approach with a microsurgical resection of the patient’s tumor was performed without significant postoperative events.

At the one-week follow-up, the patient presented with no complaints except mild postoperative dysgeusia that was described as a minimized sensation of taste. Four months after surgery, audiometry revealed an improvement in hearing function, and six months after surgery, the patient followed up with his neuro-otologist and reported improvement in his hearing, dizziness, and balance, although he did report experiencing some generalized numbness on the right side of his head. Ten months after surgery, the patient did recount some occasional tension headaches that occur approximately once per week, but he also noted that his hearing and balance on his right side had both improved.

Preoperative audiometry

**Figure 1:**
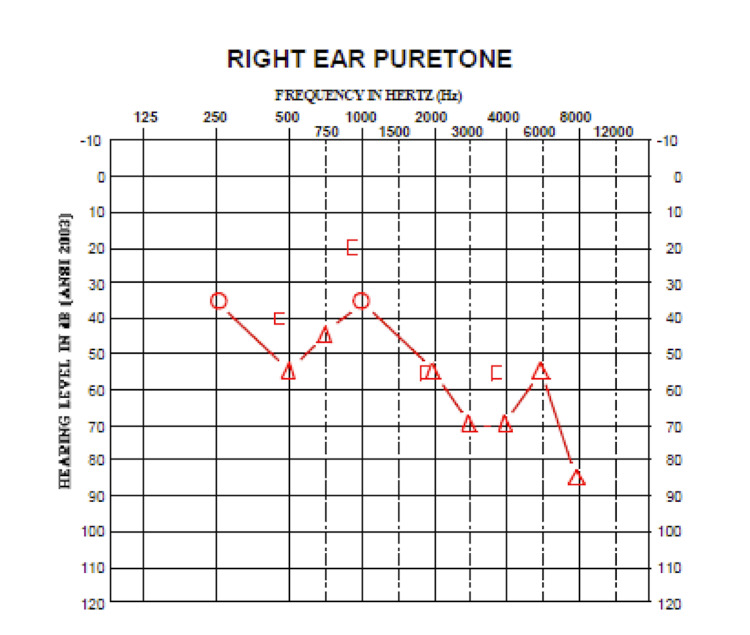
Preoperative audiogram of the afflicted side Air conductance: unmasked (circle) and masked (triangle)

Audiometry was performed approximately seven months and one week prior to surgery (Tables 1, 2 and Figure 1).** **Postoperative audiometry demonstrated an increased word recognition score in the left ear and maintenance of the operative-side speech reception thresholds.

**Table 1:** Audiometry results SR, speech reception; WR, word recognition; R, right; L, left

	7 months preoperative	1 week preoperative	4 months postoperative
SR threshold (R)	30 dB	35 dB	30 dB
SR threshold (L)	15 dB	15 dB	15 dB
WR score (R)	84%	48%	48%
WR score (L)	96%	100%	100%

**Table 2:** Preoperative hearing of the afflicted side A/C, air conductance; A/C-m, air conductance masked; B/C, bone conductance; B/C-m, bone conductance masked

Frequency (Hz)	125	250	500	750	1000	1500	2000	3000	4000	6000	8000
A/C		35			35						
A/C-M			55	45			55	70	70	55	85
B/C											
B/C-M			40		20		55		55		

Preoperative imaging

The patient underwent MRI of his brain and IAC with and without contrast approximately five months prior to surgery that revealed a heterogeneous mass (2.2 x 1.3 x 2.0 cm) in the right CPA-IAC compatible with a cystic VS. A mild mass effect was noted on the right side of the cerebellum and pons without abnormal signal or hydrocephalus.

**Figure 2:**
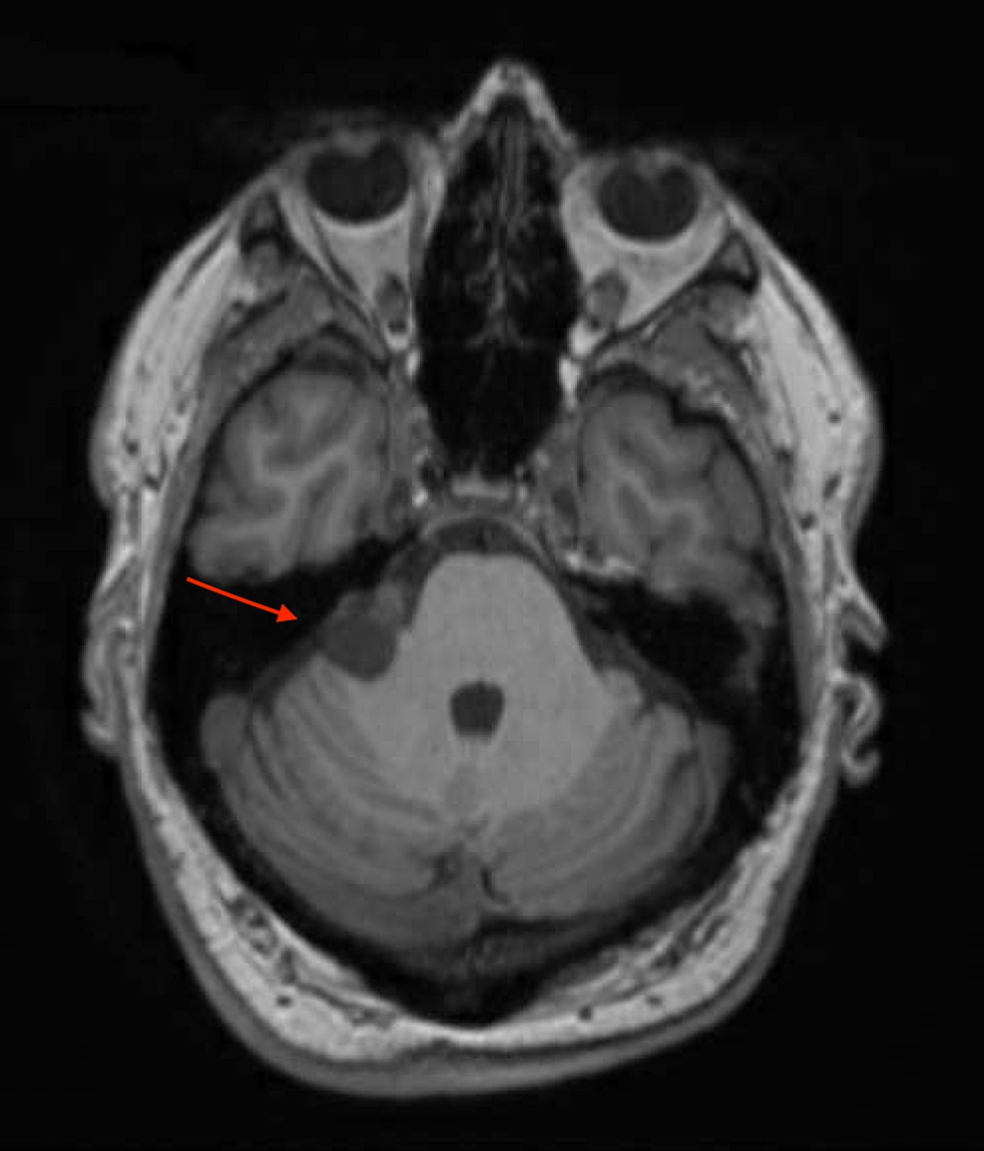
Preoperative T1-weighted MRI with contrast demonstrating right CPA mass indicated by the red arrow CPA, cerebellopontine angle

Preoperative imaging was performed approximately one month prior to surgery. A CT angiogram of his brain with contrast demonstrated contact of the right anterior inferior cerebellar artery with the anterior and medial borders of the tumor, and a brain MRI with and without contrast was also performed (Figure 2).

Surgical resection

The patient underwent a complex stereotactic extended RS craniotomy for a transcondylar posterior fossa approach with a microsurgical resection of his CPA mass. The tumor was visualized using the vertebral and anterior inferior cerebellar arteries and was dissected in its posterior, superior, and inferior borders. A small carpet of tumor was extremely adhesive to the brainstem as well as to the region of the facial nerve, and we left a thin layer of carpet of tumor on the brainstem and on the facial nerve as it extended into the IAC. The lesion was noted to be of a yellowish color with soft consistency and significant attachments. The tumor specimen was sent off to pathology, and the frozen specimen came back as schwannoma.

Postoperative audiometry

**Figure 3:**
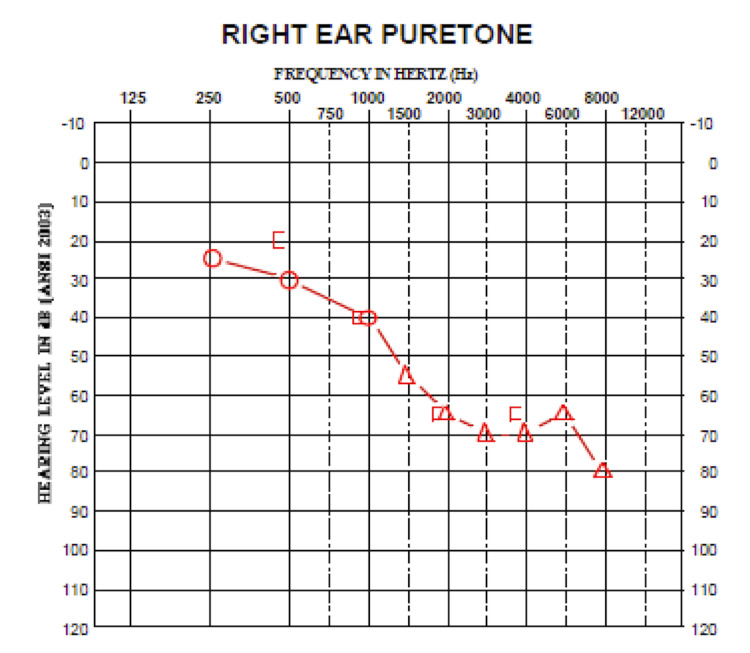
Postoperative audiogram of the afflicted side Air conductance: unmasked (circle) and masked (triangle)

Approximately four months after surgery, the patient underwent postoperative audiometry at the same center (Tables 1, 3 and Figure 3). Postoperative audiometry demonstrated an increased word recognition score for the left ear and maintenance of operative-side speech reception thresholds.

**Table 3:** Postoperative hearing of the afflicted side A/C, air conductance; A/C-m, air conductance masked; B/C, bone conductance; B/C-m, bone conductance masked

Frequency (Hz)	125	250	500	750	1000	1500	2000	3000	4000	6000	8000
A/C		25	30		40						
A/C-M						55	65	70	70	65	80
B/C											
B/C-M			20		40		65		65		

Postoperative imaging

Brain MRI with and without contrast was also performed that demonstrated postoperative changes related to the resection of the tumor, along with some possible representation of residual tumor along the right surface of the brainstem as well as the right IAC. In addition, mass effect on the right brainstem and cerebellum significantly improved compared to preoperative imaging.

**Figure 4:**
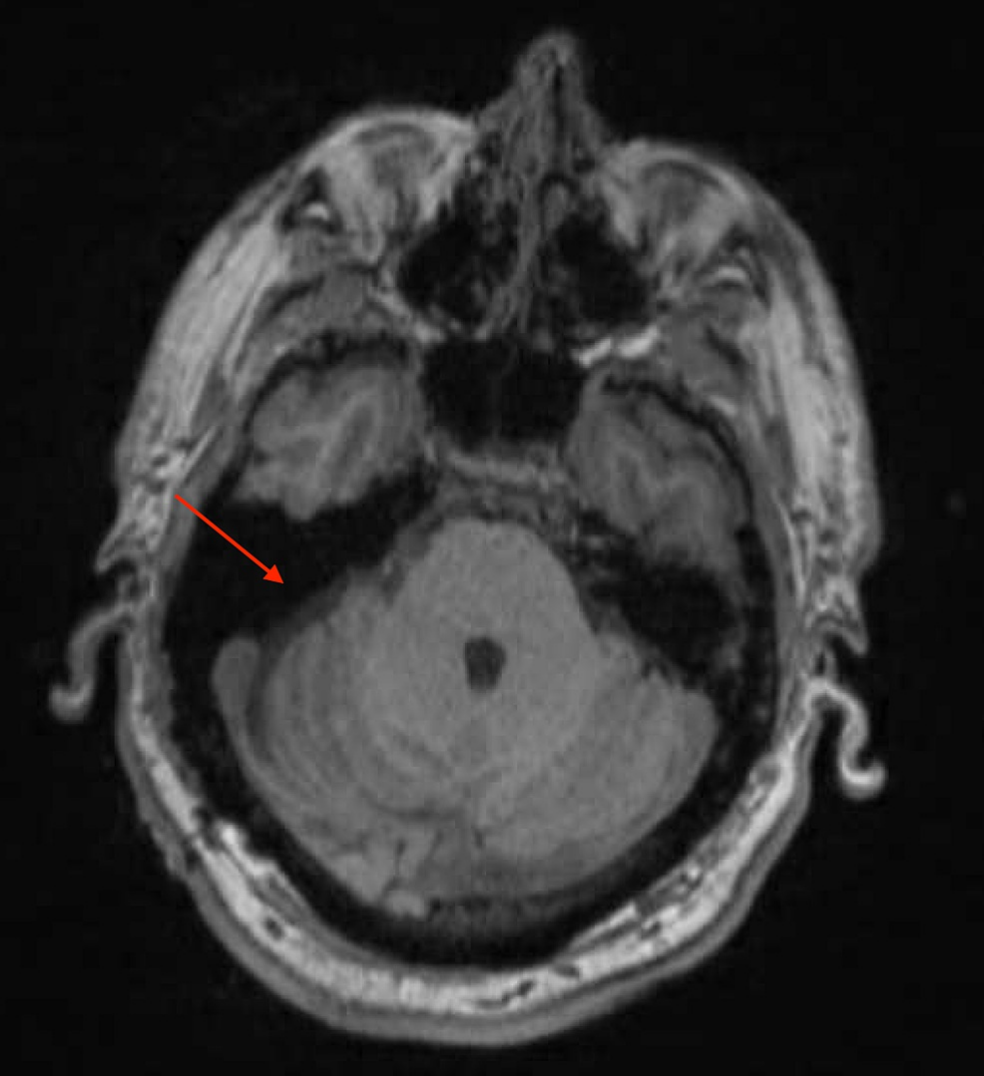
Postoperative T2-weighted MRI with contrast demonstrating postsurgical changes, with the site of resection indicated by the red arrow

Approximately 10 months after surgery, an MRI-IAC with and without contrast was performed that demonstrated stability of the residual tumor (Figure 4).

## Discussion

VS are benign intracranial neoplasms arising from the Schwann cells that line the vestibular branch of CN VIII [3]. The surgical resection of VS may be performed utilizing either a TL, MCF, or RS approach. Each has its own advantages and disadvantages in regard to tumor control and hearing preservation. In particular, the MCF approach typically allows for the best rates of hearing preservation, whereas the RS approach allows for the resection of tumors of all sizes at the possible expense of hearing function preservation [7,8]. Here, we present a VS case where the RS approach was performed and hearing function improved postoperatively.

Hearing function improvement

Hearing preservation in VS resection is typically attempted when the word recognition score is greater than 70% [7]. However, our patient’s preoperative right-sided word recognition score was 48%, and after surgery, his word recognition score improved to 66%. In addition, his right-sided speech reception threshold dropped from 35 to 30 dB. Some studies have previously suggested that preoperative hearing may not be correlated with postoperative hearing in resection of VS, and indeed, our patient’s postoperative hearing improvement supports the idea that the betterment of hearing function is still a viable goal in VS resection even if preoperative hearing function is less than ideal [9].

Tumor control

A small amount of residual tumor was intentionally left due to its strong adhesion to the brainstem and facial nerve. Upon imaging 10 months postoperatively, it was determined that this residual tumor was stable. MR imaging with gadolinium contrast media is typically used to surveil residual tumors in order to ensure the timely recognition of regrowth and recurrence [10]. One study has suggested surveillance for up to 15 years depending on the degree of initial resection, and in the event of recurrence, radiotherapy modalities such as Gamma Knife (Elekta, Stockholm, Sweden), CyberKnife® (Accuray Inc., Sunnyvale, CA, USA), and linear accelerators may be used [11-14].

## Conclusions

Although the RS approach for resection of VS is typically not associated with the highest rates of hearing preservation (47% hearing preservation via RS vs 63% hearing preservation via MCF), this case demonstrates improved postoperative hearing function in a patient whose tumor was resected via the RS approach. In addition, his residual tumor was stable at 10-month follow-up with no adverse outcomes associated with the surgery. Therefore, postoperative hearing improvement may still be attainable in VS patients with significant hearing loss, particularly with the RS approach.
